# Epithelial Cells Derived from Swine Bone Marrow Express Stem Cell Markers and Support Influenza Virus Replication *In Vitro*

**DOI:** 10.1371/journal.pone.0029567

**Published:** 2011-12-22

**Authors:** Mahesh Khatri, Yehia M. Saif

**Affiliations:** Food Animal Health Research Program, Ohio Agricultural Research and Development Center, The Ohio State University, Wooster, Ohio, United States of America; Hallym University, Republic of Korea

## Abstract

The bone marrow contains heterogeneous population of cells that are involved in the regeneration and repair of diseased organs, including the lungs. In this study, we isolated and characterized progenitor epithelial cells from the bone marrow of 4- to 5-week old germ-free pigs. Microscopically, the cultured cells showed epithelial-like morphology. Phenotypically, these cells expressed the stem cell markers octamer-binding transcription factor (Oct4) and stage-specific embryonic antigen-1 (SSEA-1), the alveolar stem cell marker Clara cell secretory protein (Ccsp), and the epithelial cell markers pan-cytokeratin (Pan-K), cytokeratin-18 (K-18), and occludin. When cultured in epithelial cell growth medium, the progenitor epithelial cells expressed type I and type II pneumocyte markers. Next, we examined the susceptibility of these cells to influenza virus. Progenitor epithelial cells expressed sialic acid receptors utilized by avian and mammalian influenza viruses and were targets for influenza virus replication. Additionally, differentiated type II but not type I pneumocytes supported the replication of influenza virus. Our data indicate that we have identified a unique population of progenitor epithelial cells in the bone marrow that might have airway reconstitution potential and may be a useful model for cell-based therapies for infectious and non-infectious lung diseases.

## Introduction

Bone marrow contains a variety of stem cells that include hematopoietic stem cells, mesenchymal stem cells or stromal cells (MSC), and multipotent adult progenitor cells [1]. Many reports using a variety of animal models have demonstrated that bone marrow cells (BMCs) may have a role in the repair and regeneration of injured lung, infarcted myocardium, and damaged bone, tendon and cartilage [2], [3], [4], [5], [6], [7], [8]. BMCs cultured *in vitro* can differentiate into type I, II, and basal and airway epithelial cells and express the cystic fibrosis transmembrane conductance regulator (CFTR) protein [9]. BMCs have been shown to improve survival and attenuate lung inflammation in bleomycin- and endotoxin-induced lung injury [8], [10], [11], [12]. Following infusion of BMCs in animal models, these cells have been identified as type I and II alveolar epithelial cells, endothelial cells, fibroblasts, and bronchial epithelial cells [12]. However, precise identity of specific subpopulation of BMCs that engraft in the lung parenchyma and have regenerative potential is still not clear.

Recently, Wong and colleagues [13] reported the isolation of progenitor epithelial cells from mouse and human bone marrow. These cells expressed Clara cell secretory protein (Ccsp), a marker of airway progenitor cells [14], CD45 and mesenchymal markers CD73, CD90, CD105. These cells differentiated into multiple epithelial cell lineages, including type I and II pneumocytes *in vitro*. Furthermore, these progenitor epithelial cells preferentially homed to naphthalene-injured lung following intratracheal or systemic inoculation.

Influenza viruses belong to the family *Orthomyxoviridae* and cause highly contagious respiratory infections in humans and animals. These viruses cause seasonal epidemics and infrequent pandemics in humans. Seasonal influenza epidemics are responsible for between 200,000 and 500,000 influenza-related deaths each year [15]. Avian influenza viruses caused three human pandemics during the last century. The 2009 pandemic, the first pandemic of 21^st^ century, was caused by a triple reassortant H1N1 influenza virus of swine lineage [16]. In addition to seasonal and pandemic viruses, highly pathogenic avian influenza (HPAI) H5N1 virus has crossed species barrier to infect humans. As of August 9, 2011, more than 500 human cases with over 300 deaths have been reported worldwide [17]. H5N1 viruses replicate to higher titers in lungs and extra-pulmonary tissues leading to acute respiratory distress syndrome, multiple-organ dysfunction, lymphopenia, and hemophagocytosis [18], [19], [20]. Influenza viruses, therefore, pose a constant public health threat, and it is important to understand its pathogenesis to devise effective control measures.

Swine are gaining popularity as a useful large animal model for stem cell therapy for important human diseases or conditions such as myocardial infarction, diabetes, atherosclerosis, traumatic brain injury, retinal damage, and tooth regeneration [21], [22], [23], [24], [25]. Like humans, pigs are an outbred species. As well, they are similar to humans in anatomy, physiology, and immune responses [26], [27], [28], [29]. Additionally, swine can serve as an excellent animal model for influenza virus pathogenesis studies. The clinical manifestations and pathogenesis of influenza in pigs closely resemble to what is observed in humans. Furthermore, the cytokine responses in branchoalveolar lavage (BAL) fluid from swine influenza virus-infected pigs are also identical to that observed in nasal lavage fluids of experimentally infected humans [30]. These observations support that pigs serve as an excellent animal model to study the pathogenesis of influenza virus [31].

In this study, we report the isolation of previously undocumented progenitor epithelial cells in pig bone marrow that expressed Clara cell secretory protein (Ccsp), a marker for lung progenitor cells, and the stem cell markers octamer-binding transcription factor (Oct4) and stage-specific embryonic antigen-1 (SSEA-1). These progenitor cells showed increased self-renewal capacity and expressed epithelial cell markers such as pan-cytokeratin (Pan-K), cytokeratin 18 (K-18), and occludin. Importantly, these cells expressed receptors for both mammalian and avian influenza viruses and were permissive to infection with these viruses. The progenitor cells differentiated into type I and II pneumocytes and type II pneumocytes also supported replication of influenza virus. These data provide new insights into the pathogenesis of influenza virus. Further, porcine progenitor epithelial cells described here may serve as a useful model in cellular therapy strategies for epithelial diseases.

## Results

### Characteristics of the progenitor epithelial cells

**Figure 1:**
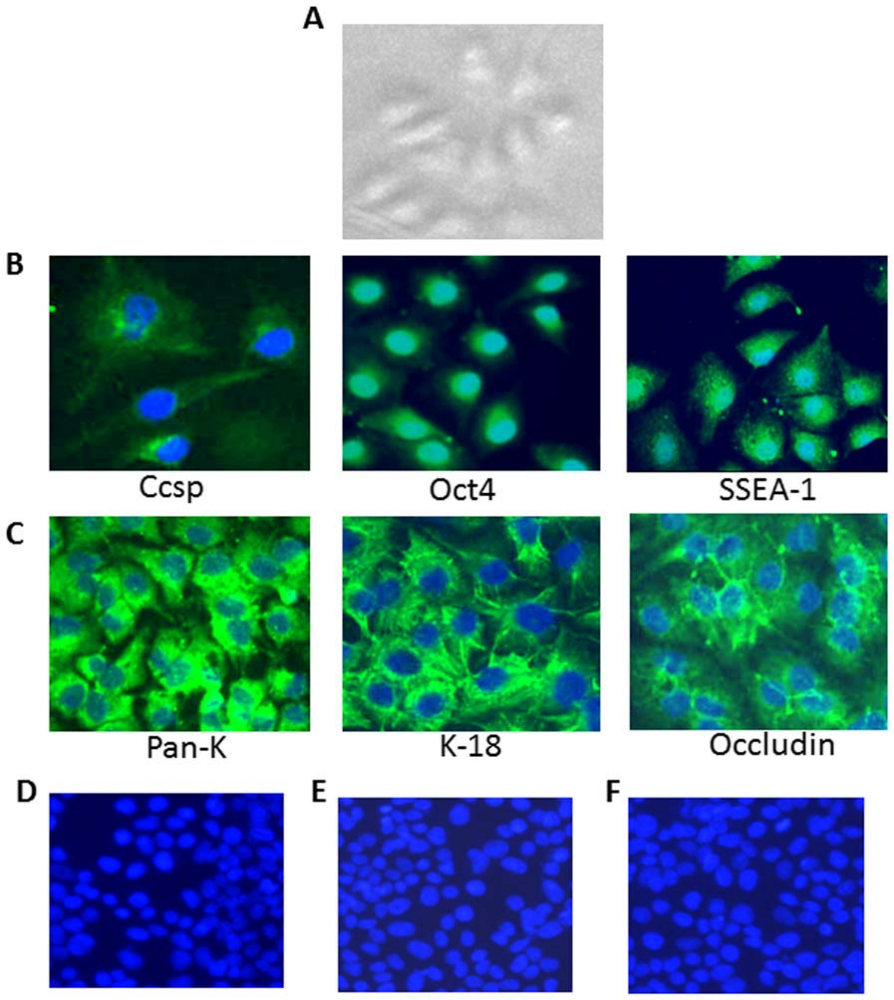
Characterization of progenitor epithelial cells isolated from pig bone marrow. (**A**) Colony morphology of progenitor epithelial cells derived from bone marrow. (**B**) Expression of stem cells markers on progenitor epithelial cells. Epithelial colony cells from primary cell cultures were examined for the expression of lung and other stem cell markers by using specific antibodies directed against Ccsp, Oct4 and SSEA-1. (**C**) Expression of epithelial cell markers on progenitor epithelial cells. Primary cell cultures were examined for the expression of specific epithelial cell markers: Pan-K, K-18, and occludin. (**D–F**) Absence of non-specific binding by progenitor epithelial cells. The cells were incubated with FITC-labeled secondary antibodies (D) goat anti-rabbit (E) goat anti-mouse (F) donkey anti-goat antibodies. Nuclei were stained by DAPI.

During the culture of BMCs for the isolation of mesenchymal stromal cells (MSC), we observed colonies of epithelial cells surrounded by mesenchymal cells. The colony cells exhibited cuboidal morphology typical of epithelial cells (Fig. 1A).

To characterize epithelial colony cells, we expanded individual colonies *in vitro* and performed immunocytochemistry by using a panel of stem cell and epithelial cell-specific antibodies. We found that the epithelial colony cells expressed Ccsp, Oct4, and SSEA-1 (Fig. 1B). We detected the expression of Oct4 in the nuclei, whereas SSEA-1 was mainly found on the cell surface and in the cytoplasm of the progenitor epithelial colony cells (Fig. 1B). The colony cells expressed epithelial specific markers Pan-K, K-18, and occludin (Fig. 1C) but not the mesenchymal markers CD29, CD44, and CD90 (data not shown).

### Differentiation potential of progenitor epithelial cells

**Figure 2:**
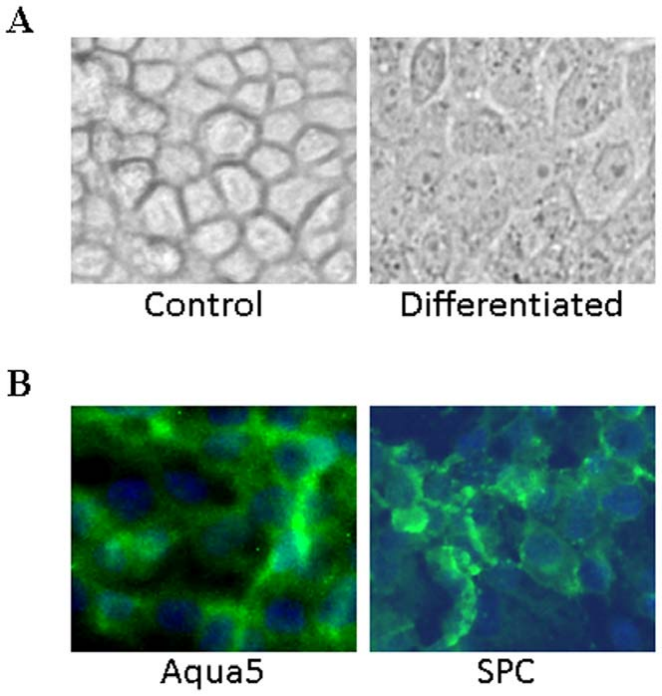
Differentiation potential of progenitor epithelial cells. Epithelial colony cells were sub-cultured onto collagen I-coated plates and cultured in epithelial cell differentiation medium; on day 5 the cells were examined for (**A**) morphology of control and differentiated cells and (**B**) the expression of alveolar cell markers, Aqua5 (a type I pneumocyte marker) and SPC (a type II pneumocyte marker).

To address the self-renewal and differentiation potential of these progenitor epithelial cells, individual colonies were isolated from primary cultures and seeded in tissue culture plates precoated with collagen I in 50% epithelial growth medium and 50% MSC conditioned medium (MSC-CM); referred to as epithelial differentiation medium in the text. In epithelial differentiation medium, the cells continued to grow, became flattened, and appeared as thinly spread cell clusters (Fig. 2A). By day 5, the expression of pro surfactant protein C (SPC) was detected in the cytoplasm of these flattened cells (Fig. 2B). These features are consistent with the type II pneumocytes. Also, expression of aquaporin 5 (Aqua5) protein, a marker for type I pneumocytes was detected on expanded cells which were significantly larger than that of the parental primary epithelial colony cells (Fig. 2B). The expression of SPC or Aqua5 was not detected on primary undifferentiated cells. These results suggest that bone marrow progenitor epithelial cells have the potential to differentiate into type I and II like pneumocytes.

### Detection of α-2,3- and α-2,6-linked sialic acid receptors on progenitor epithelial cells

**Figure 3:**
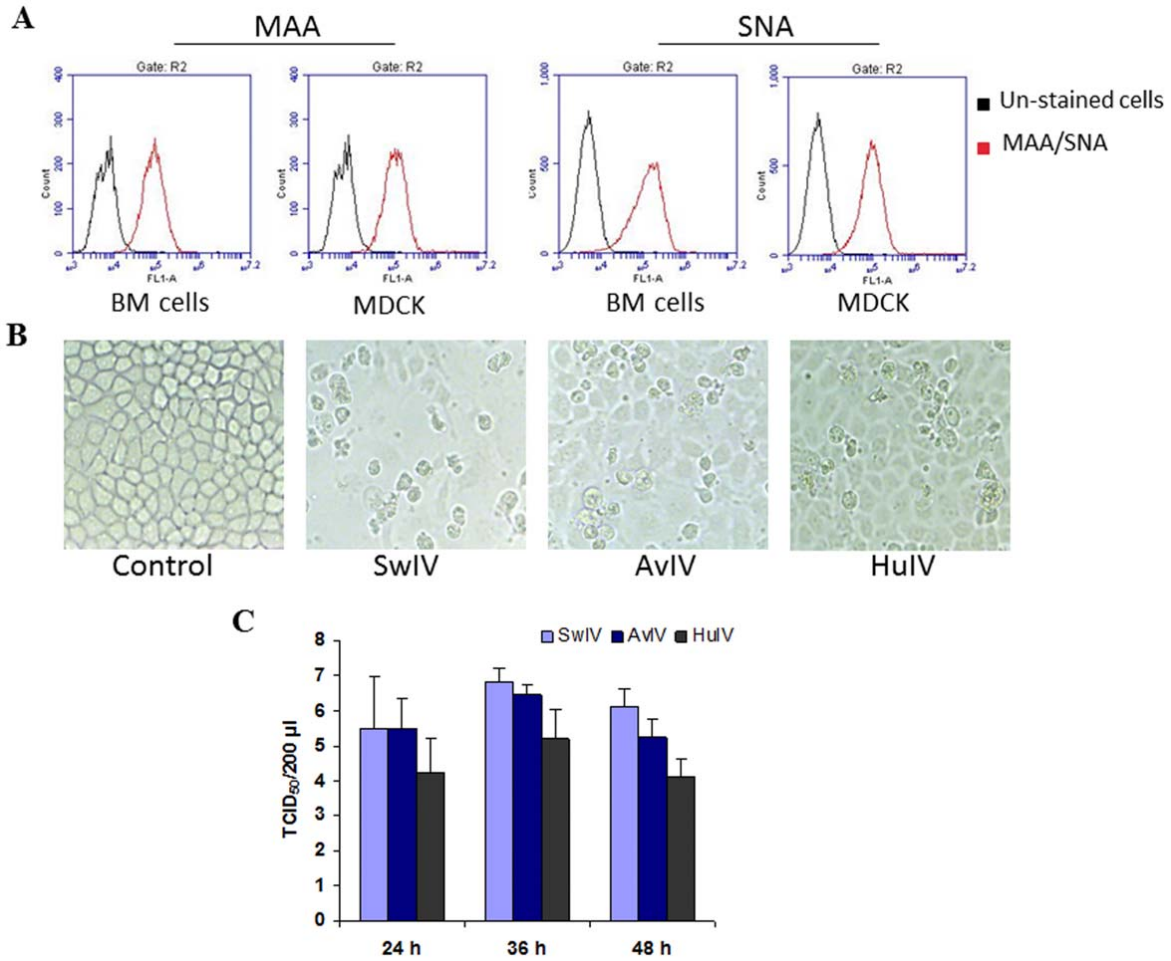
Replication of influenza virus in progenitor epithelial cells. (**A**) Progenitor epithelial cells express α-2,3- and α-2,6-linked sialic acid receptors. Progenitor epithelial cells were examined by flow cytometry for the expression of α-2,3- and α-2,6-linked sialic acid receptors. The cells were stained with FITC-labelled *Maackia amurensis* lectin II (MAA) specific for α-2,3-linked sialic acid receptors and *Sambucus niagra* agglutinin (SNA), specific for α-2,6-linked sialic acid receptors. MDCK cells were included as positive controls. Compared to unstained cells (black), cells stained with lectins (red) showed right shift indicating positive staining. BM cells (progenitor bone marrow epithelial cells), MDCK (MDCK cells) (**B**) Progenitor epithelial cells were infected with SwIV, AvIV or HuIV at a MOI of 1. All virus types tested induced cell cytotoxicity. (**C**) Virus replication kinetics in primary culture of progenitor epithelial cells after virus infection at 1 MOI. Virus production in infected culture supernatants was measured by titration in MDCK cells. The values are averages ± S.D. of two separate experiments using progenitor epithelial cells from two different pigs in each experiment (n = 4).

Influenza virus infects cells through binding to cell surface sialic acid receptors. To examine the susceptibility of progenitor epithelial cells to influenza virus, we first evaluated by flowcytometry these cells for the presence of sialic acid receptors. The α-2,3- and α-2,6-linked sialic acid receptors were detected on the surface of progenitor epithelial cells by lectin staining. A majority of progenitor cells (>95%) expressed both α-2,3- and α-2,6-linked sialic acid receptors, indicating that viruses of both avian and mammalian lineages might replicate in these cells (Fig. 3A).

### Progenitor epithelial cells as targets for influenza virus replication

After confirming the expression of sialic acid receptors on progenitor epithelial cells, we next examined the susceptibility of progenitor epithelial cells to mammalian and avian influenza viruses. Cells were inoculated with SwIV, AvIV or HuIV at a MOI of 1 for 1 hour (h). As shown in Fig. 3, infectious viruses were observed^ in^ culture supernatants after 24 h after infection and viral titers slightly increased at 36 h. Among the viruses tested, SwIV produced highest cellular lysis (Fig. 3B) and replicated to highest titers followed by AvIV and HuIV (Fig. 3C). The production of cytopathic effects required live, infectious virus because cell cytotoxicity was not observed in heat-inactivated virus-infected cultures (data not shown).

**Figure 4:**
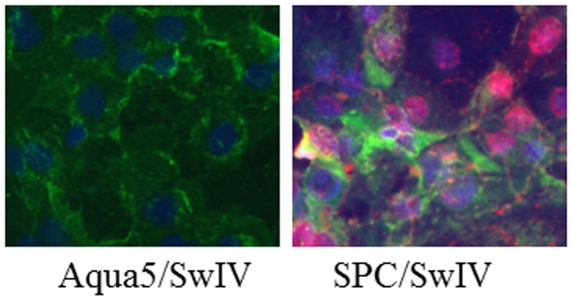
Replication of influenza virus in differentiated pneumocytes: Primary cells were subcultured onto collagen I-coated plates in epithelial cell differentiation medium; on day 5 differentiated cells were infected with SwIV and examined for the presence of viral proteins at 24 h after infection. Type II pneumocytes, positive for SPC marker (green) supported the replication of SwIV virus as indicated by expression of viral NP protein (red). Cell nuclei were stained with DAPI (blue). No viral proteins were detected in Aqua5 expressing (green) type I pneumocytes.

In addressing whether *in vitro* differentiated type I and II pneumocytes are susceptible to influenza virus infection, progenitor cells were differentiated to type I and II pneumocytes for 5 days in epithelial differential medium. The differentiated cultures were then exposed to SwIV. Co-immunostaining revealed SwIV replication in differentiated type II pneumocytes as indicated by the presence of viral proteins whereas no viral proteins were detected in differentiated type I pneumocytes (Fig. 4).

## Discussion

In this study, we report the isolation and identification of previously undocumented stem/progenitor epithelial cells from bone marrow of pigs. These cells expressed the stem/progenitor cell markers Oct4, SSEA-1, and Ccsp, and the epithelial markers Pan-K, K-18, and occludin. Upon culture in epithelial differentiation media, these cells differentiated into type I and II pneumocytes. Most importantly, progenitor cells were targets for mammalian and avian influenza virus replication.

We detected the expression of Oct4 in progenitor epithelial cells. Oct4, a member of the POU family of transcription factors, is expressed in pluripotent stem cells such as embryonic stem cells, induced pluripotent stem cells, and lung stem cells where it regulates self-renewal and pluripotency [32], [33]. These Oct4^+^ colony cells also expressed other stem cell markers; SSEA-1, Ccsp and epithelial markers; pan-K, K-18, and occludin. Clara cells, airways progenitor cells in the lungs which have been involved in lung regeneration and repair, also express Ccsp and cytokeratins [14], [32], [34]. Importantly, these Oct4^+^Ccsp^+^ colony cells differentiated into cells possessing Aqua5 and SPC which are markers for type I and II like pneumocytes respectively. Expression of stem cell markers in progenitor epithelial cells may, therefore, be associated with differentiation potential of these cells.

The porcine progenitor epithelial cells reported in this study share similarities with recently reported human and mouse cells that both cell types express Ccsp, but unlike human and mouse cells, porcine progenitor epithelial cells did not express mesenchymal markers such as CD29, CD44 and CD90 [13]. Porcine progenitor epithelial cells also expressed stem cell markers; Oct4 and SSEA-1; however, mouse and human cells were not tested for expression of these markers [13]. Similar to human and mouse bone marrow cells, porcine bone marrow progenitor cells expressed both Ccsp and SPC. Ccsp and SPC expressing broncho-alveolar stem cells (BACS) were recently identified in adult mouse lung [35]. Mouse BACS were found to express hematopoietic markers, whereas our porcine bone marrow progenitor epithelial cells lacked the expression of these markers. Importantly, we were able to passage progenitor epithelial cells up to passage 8 suggesting that these cells have extensive self-renewal and proliferation properties.

The role of bone marrow stem cells in tissue regeneration including lungs is well established [36], [37], [38], [39]. However, the precise identity of specific subpopulation of BMCs that has tissue regeneration capacity is not known. As reported for human progenitor epithelial cells, porcine Oct4^+^ Ccsp^+^ colony cells were detected in the plastic-adherent fraction surrounded by mesenchymal cells [40]. Therefore, it is likely that the progenitor epithelial cells were derived from mesenchymal precursors. Previously, bone marrow and even cord blood MSC were shown to have epithelial differentiation potential. Cord blood MSC when cultured *in vitro* expressed lung-specific markers such as Ccsp, SPC, and CFTR [41]. Intravenous administration of cord blood-MSC into NOD-SCID mice resulted in a low level of airway engraftment of the cord blood-MSC that expressed cytokeratin and CFTR. Similarly, bone marrow MSC differentiated to type I and II pneumocytes *in vitro* and following intravenous or intratracheal administration, engrafted in recipient lung parenchyma as type I and II pneumocytes [3], [42]. These observations suggest that bone marrow MSC could serve as precursors of differentiated epithelial cells. Moreover, in this study we observed that progenitor epithelial cells when cultured in 50% MSC-CM were able to differentiate into type I and II pneumocytes further providing evidence that mesenchymal stroma is important for the pluripotency of epithelial colony cells. Future work will be directed to identify the molecular signals provided by the mesenchymal precursors that regulate the differentiation potential of epithelial progenitor cells.

Bone marrow cell therapy may be effective in conditions that currently lack effective treatment. Stem cells isolated from different anatomic niches of lungs [34], [35], [43], [44], [45] have been shown to differentiate into multiple epithelial cell types following lung injury. Our lab and Wong and colleagues [13] demonstrated the existence of progenitor epithelial cells in the bone marrow of pigs and humans respectively which are capable of differentiating into type I and II pneumocytes suggesting that these cells will be valuable for lung therapies. Functionally, type I pneumocytes are important for gas exchange, water permeability and the regulation of alveolar fluid homeostasis whereas type II pneumocytes produce lung surfactants that reduce the alveolar surface tension [46]. The isolation and expansion of lung-derived autologous cells from humans is difficult, whereas bone marrow progenitor cells can be easily isolated and expanded. In other preliminary studies, we have isolated progenitor epithelial cells from the lung of pigs. Future *in vivo* experiments in pigs will be designed to compare the lung repair potential of bone marrow- and lung-derived progenitor epithelial cells following infection and/or LPS-induced lung injury.

Excessive virus replication, multi-organ failure, and hyperimmune activation have been detected in humans and laboratory animals infected with HPAI H5N1 and with recreated 1918 pandemic influenza virus [47], [48], [49], [50], [51]. The mechanisms responsible for deterioration of lung function and the loss of capacity for lung repair after influenza virus infection are not well understood. Although influenza virus primarily replicates in lung, virus has been detected in extra pulmonary tissues including bone marrow [52], [53]. Our *in vitro* findings show that progenitor epithelial cells in bone marrow are permissive to influenza virus suggesting that in the event of influenza virus infection, bone marrow progenitor cells may not be available to home to the injured lung to participate in tissue repair. Additional experiments are needed to confirm the infection of progenitor epithelial cells *in vivo* and to delineate further their roles in local and lung repair following infection with influenza virus.

In conclusion, we have isolated Oct4^+^ Ccsp^+^ SSEA-1^+^ expressing progenitor epithelial cells in the bone marrow of swine which are capable of differentiating into type I and II pneumocytes. Additionally, we have demonstrated that these cells serve as targets for influenza virus infection. Further characterization of these progenitor cells may help in understanding the pathogenesis of infectious and non-infectious epithelial diseases and the mechanisms of lung injury repair.

## Materials and Methods

### Isolation and identification of bone marrow progenitor epithelial cells

Femur bones were obtained from 4- to 6-week old germ-free pigs. BMCs were isolated by previously described methods [54], [55], [56]. Briefly, the tip of each bone was removed and the marrow was harvested by inserting a syringe needle into one end of the bone and flushing with Dulbecco's Modified Eagle's Medium (DMEM; Gibco). The bone marrow cells were filtered through a 70-µm nylon mesh filter (BD, Falcon, USA) and mononuclear cells were obtained by density gradient centrifugation over Ficoll-Hypaque (gradient density 1.077). Cells (1–5×10^5^/cm^2^) were plated in 25 cm^2^ cell culture flasks in DMEM containing 10% fetal bovine serum, 2 mm L-glutamine, 1% antibiotic solution (Gibco, USA). Cultures were incubated at 37°C in a humidified atmosphere containing 95% air and 5% CO_2_. The non-adherent cells were removed after 72 h of culture. Individual epithelial colonies surrounded by mesenchymal cells were plucked and were expanded *in vitro* by culturing in DMEM media.

### Detection of stem cell and epithelial cell markers

Stem cell and epithelial cell markers on progenitor epithelial cells were detected by immunofluorescence assay (IFA). Epithelial colony cells were fixed in methanol: acetone (1∶1) for 3 min at room temperature and then blocked with 3% bovine serum albumin (BSA) for 30 min. Cells were incubated at 4°C with following primary antibodies: rabbit anti-human Oct4 (Santa Cruz Biotechnology), mouse anti- human SSEA-1(Millipore), goat anti-mouse cc10 (Santa Cruz Biotechnology), mouse anti-human pan cytokeratin, mouse anti-human cytokeratin 18 (Sigma), rabbit anti-human occludin (Invitrogen), mouse anti-human CD90, rat anti-mouse CD44 and mouse anti-pig CD29 (BD Pharmingen). After overnight incubation at 4°C, cells were washed and incubated for 1 h at room temperature with the following respective FITC-labeled secondary antibodies: donkey anti-goat IgG, goat anti-rabbit IgG, and goat anti-mouse IgG (Sigma). Cell nuclei were then counterstained with 4′,6-diamidino-2-phenylindole (DAPI).

### Generation of bone marrow MSC-CM

Porcine bone marrow MSC were grown and expanded as described above. When MSC reached at about 80% confluence, medium was aspirated and cells were washed three times with phosphate-buffered saline. Cells were cultured in serum-free medium for 24 h. CM was removed and centrifuged to remove cellular debris and used in epithelial differentiation assays.

### Differentiation of bone marrow progenitor epithelial cells into type I and type II pneumocytes

To analyze differentiation potential of porcine progenitor epithelial cells, cells were seeded in culture dishes pre-coated with collagen I. The cells were cultured in epithelial cell differentiation medium containing 50% epithelial growth media supplemented with bovine pituitary extract (70 µg/ml), human epidermal growth factor (5 ng/ml), insulin (5 µg/ml), and hydrocortisone (0.5 µg/ml) (MEGM, Lonza, Walkersville, MD) and 50% MSC-CM medium. After 5 days of incubation at 37°C, cells were observed for morphology and cells grown on coverslips were examined for the expression of Aqua5 (marker for type I pneumocytes) and SPC (marker for type II pneumocytes) by using goat anti-human Aqua5 (Santa Cruz Biotechnology) and rabbit anti-human SPC (Millipore) primary antibodies by IFA as described above.

### Detection of sialic acid receptors on progenitor epithelial cells

Sialic acid receptors on progenitor epithelial cells were detected by flowcytometry [57]. Madin-Darby canine kidney (MDCK) cells have been shown to express both α-2,3-linked sialic acid receptors and α-2,6-linked sialic acid receptors; avian and mammalian influenza virus receptors, respectively. Therefore, MDCK cells were included as positive controls. The cells were incubated with FITC-labeled *Maackia amurensis* lectin II (MAA) or *Sambucus niagra agglutinin* (SNA) (EY Laboratories) at 4°C for 30 min in dark and acquired by Accuri C6 flow cytometer (BD Accuri) and analyzed using CFlow plus software (Accuri).

### Viruses and infection of progenitor epithelial cells

Influenza virus strains; swine influenza virus (SwIV; Sw/OH/07, H1N1), avian influenza virus (AvIV; Ch/NY/95, H7N2) and human influenza virus (HuIV; Hu/OH/06, H1N1) were propagated in 10-day-old embryonated chicken eggs (AvIV was kindly provided by Dr. S.M. Goyal, Department of Veterinary Population Medicine, University of Minnesota, Saint Paul, MN). Progenitor epithelial cells were infected with SwIV, AvIV and HuIV at a multiplicity of infection (MOI) of 1. After adsorption for 1 h, the cells were washed and fresh medium was added to the cells. At intervals after infection, released viruses in culture supernatants were titrated in MDCK cells [58].

To examine whether differentiated progenitor epithelial cells support influenza virus infection, epithelial colony cells were cultured in collagen I coated dishes for 5 days in epithelial cell differentiation medium. On day 5, cells were infected with SwIV and influenza viral nucleoprotein (NP) was detected at 24 h after infection by IFA using mouse anti influenza A NP (Millipore) as primary antibody and rhodamine-labelled goat anti-mouse (Molecular Probes) as secondary antibody.
